# Screening of the Active Component Promoting Leydig Cell Proliferation from *Lepidium meyenii* Using HPLC-ESI-MS/MS Coupled with Multivariate Statistical Analysis

**DOI:** 10.3390/molecules24112101

**Published:** 2019-06-03

**Authors:** Xiao-chen Gao, Jing-wei Lv, Chun-nan Li, Nan-xi Zhang, Lin-lin Tian, Xi-ying Han, Hui Zhang, Jia-ming Sun

**Affiliations:** 1 Jilin Ginseng Academy, Changchun University of Chinese Medicine, Changchun 130117, Jilin, China; gao_xiaochen@hotmail.com (X.-c.G.); jingwei-lv@hotmail.com (J.-w.L.); lcn1013@hotmail.com (C.-n.L.); nancy_8080@outlook.com (N.-x.Z.); tll19900312@163.com (L.-l.T.); 2 College of traditional Chinese Medicine, Changchun University of Chinese Medicine, Changchun 130117, Jilin, China; hanxy385@nenu.edu.cn

**Keywords:** *Lepidium meyenii*, high-performance liquid chromatography-electrospray ionization/mass spectrometry, partial least squares, ultrafiltration affinity, molecular docking

## Abstract

*Lepidium meyenii* is now widely consumed as a functional food and medicinal product, which is known as an enhancer of reproductive health. However, the specific chemical composition and mechanism of action for improving sexual function are unclear. The present study aims at screening and determining the potential compounds, which promote mouse leydig cells (TM3) proliferation. The partial least squares analysis (PLS) was employed to reveal the correlation between common peaks of high performance liquid chromatography (HPLC) fingerprint of *L. meyenii* and the proliferation activity of TM3. The results suggested that three compounds had good activities on the proliferation of TM3 and promoting testosterone secretion, there were *N*-benzyl-hexadecanamide, *N*-benzyl-(9z,12z)-octadecadienamide and *N*-benzyl-(9z,12z,15z)-octadecatrienamide which might be the potential bioactive markers related to the enhancing sexual ability functions of *L. meyenii*. The first step in testosterone synthesis is the transport of cholesterol into the mitochondria, and the homeostasis of mitochondrial function is related to cyclophilin D (CypD). In order to expound how bioactive ingredients lead to promoting testosterone secretion, a molecular docking simulation was used for further illustration in the active sites and binding degree of the ligands on CypD. The results indicated there was a positive correlation between the binding energy absolute value and testosterone secretion activity. In addition, in this study it also provided the reference for a simple, quick method to screen the promoting leydig cell proliferation active components in traditional Chinese medicine (TCM).

## 1. Introduction

*Lepidium meyenii* (Maca), is a Brassicaceae *Lepidium* plant native to the Andes Mountains of South America. It has been traditionally used as a food and machine over 5000 years [1]. As is usual with many traditional folk medicines, many claims have been made regarding the efficacy of Maca in treating a wide range of illnesses and medical conditions [2,3]. However, in the 20th century most of the scientific attention has been focused in the areas where the pharmacological actions of Maca seem most strongly attested, these include, enhancement of sexual drive in humans, increasing overall vigour and energy levels, and increasing sexual fertility in humans and domestic livestock [3]. *Lepidium meyenii* is rich in nutrients and secondary metabolites with a variety of biological activities. Its main chemical compositions are polysaccharide, flavone, saponin, microelement and amino acid [4]. Low polarity magamide is considered to be its unique iconic ingredient, at present, the method of solvent reflux, ultrasonic extraction, high performance liquid chromatography (HPLC) and liquid chromatography mass spectrometry were used to detect it [5].

The present studies abroad have been studying the pharmacological effects of *L. meyenii*, they focus mainly on the effect of sexual function in mice. However all of these studies are segmentary, and lack of a comprehensive and systemic assessment, as well as the effect mechanism of improving sexual function is not yet clear. Especially, there is no research on the *L. meyenii* active monomers in promoting the mechanism of sexual function [6,7,8].

Testosterone is a prerequisite for normal spermatogenesis. Leydig cells are the main cells responsible for the production and secretion of the testosterone hormone [9]. The raw material for testosterone synthesis is cholesterol. The rate-limiting enzyme steroidogenic acute regulatory protein (StAR) in testosterone synthesis is responsible for accelerating the transport of cholesterol to the mitochondria, which is the first step in testosterone biosynthesis. For the maintenance of the StAR function, the homeostasis of the mitochondrial function is indispensable. In the process of maintaining mitochondrial function homeostasis, CypD plays an important regulatory role. Activation of CypD leads to opening of the mitochondrial permeability transition pore (mPTP) on the outer membrane of mitochondria which causes mitochondrial damage [10,11]. Mitochondrial dysfunction results in the inhibition of StAR expression, hindering cholesterol from entering the mitochondrial stromal membrane and inhibiting testosterone secretion; the CypD inhibitor can effectively bind CypD and inhibit the *cis*-*trans* isomerase activity of CypD, making the StAR expression stable, ultimately promoting testosterone secretion. Although the complete mechanism of the mPTP opening remains unclear, cyclosporine A (CsA), a high-affinity cyclophilin inhibitor, blocks the mPTP opening by binding to the CypD [12,13,14,15,16].

**Figure 1:**
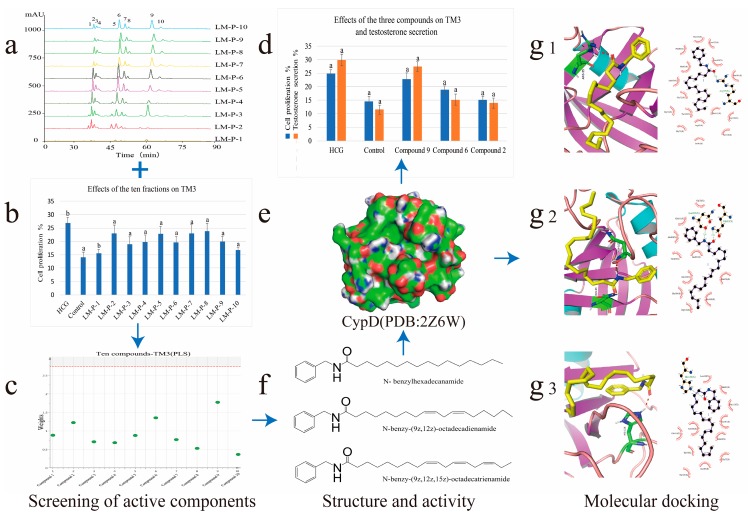
Strategy based on high-performance liquid chromatography-electrospray ionization/mass spectrometry (HPLC-ESI-MS/MS) coupling with the multivariate statistical analysis method to screen and identify the bioactive ingredients for the proliferation of mouse leydig cells (TM3) and promoting testosterone secretion in *Lepidium meyenii*. Molecular docking was used to investigate the mechanism of bioactive compounds. (**a**) The HPLC fingerprints of ten fractions. (**b**) Effects of the ten fractions on TM3 (a *p* < 0.01, b *p* < 0.05). (**c**) Model effect weights of the ten compounds on TM3. (**d**) Effects of the three compounds on TM3 and testosterone secretion (a *p* < 0.01). (**e**) The crystal structure of human cyclophilin D (PDB ID: 2Z6W). (**f**) The chemical structures of three bioactive markers. (**g_1_**) Molecular docking of compound (**9**) with CypD showed in three-dimensional (3D) and two-dimensional (2D). (**g_2_**) Molecular docking of compound (**6**) with CypD showed in 3D and 2D. (**g_3_**) Molecular docking of compound (**6**) with CypD showed in 3D and 2D.

Inspired by the applications mentioned above, in order to find out the bioactive markers reflecting the traditional efficacy, an effective strategy on the high-performance liquid chromatography-electrospray ionization/mass spectrometry (HPLC-ESI-MS/MS) coupling with multivariate statistical analysis was developed to screen and identify the bioactive ingredients in *L. meyenii* [17]. Molecular docking was used to investigate the mechanism of bioactive compounds for improving sexual function, as depicted in Figure 1. The present study illustrated and explained the underlying correlations between active constituents and mechanisms of action [18].

## 2. Results and Discussion

### 2.1. High-Performance Liquid Chromatography-Photodiode Array Detector-Electrospray Ionization/Mass Spectrometry Method Analysis of Ten Common Peeks

**Figure 2:**
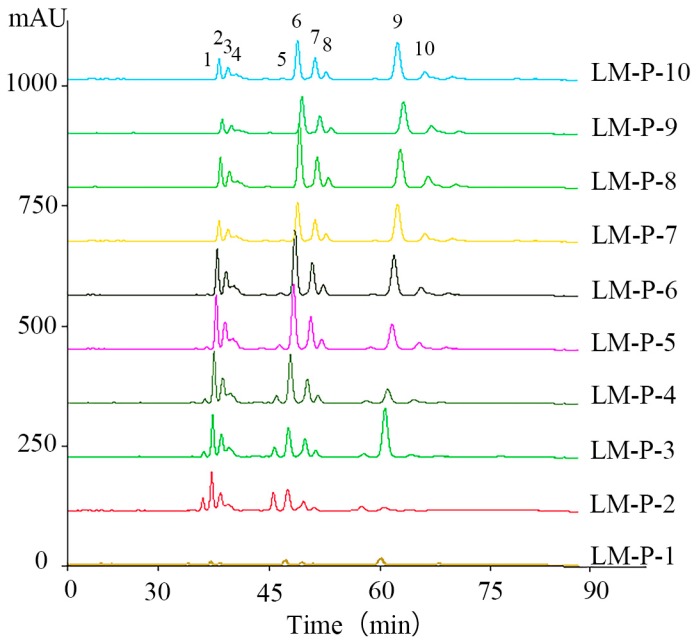
The HPLC fingerprints of ten fractions.

Ten compounds (**1**)–(**10**) were found in fraction LM-P-1 to fraction LM-P-10 at the characteristic wavelength of 210 nm. All the constituents of 10 fractions were separated and detected within 80 min and their MS^2^ data were detected in a positive ion mode (Figure 2) according to the research of fragmentation pathway for compositions of 10 fractions in electrospray ionization using MS2 ion trap mass spectrometry and comparing retention time. Their structures were elucidated based on the analyses of ultraviolet (UV) spectra and ESI–MS2 fragmentation patterns with those of standards and the corresponding spectroscopic data given in the literatures.

**Figure 3:**
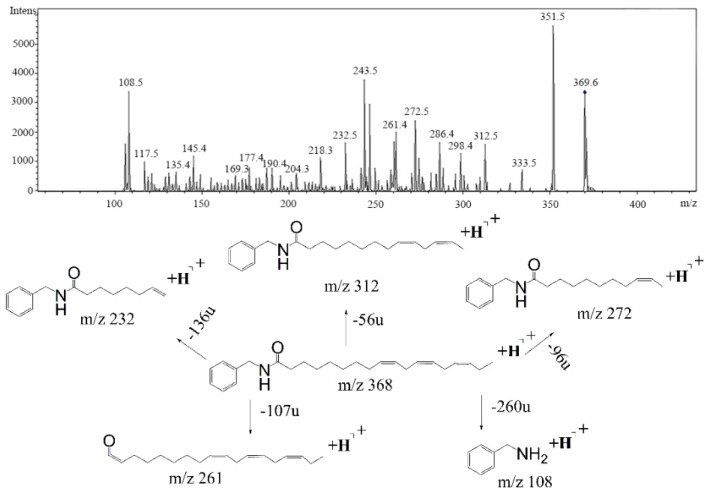
The collision-induced dissociation (CID) spectra of a *N*-benzyl-(9z,12z,15z)-octadecatrienamide.

An overview of identified compounds was shown in Table 1. Meanwhile, the detailed structural analysis of common peaks was taken as an example to illustrate that our paradigm in this part bore out the correctness of structural presumption by using MS2. The Fragmentation mode were consistent with the previously reported, it suggested that Compound (**2**) of m/z 368 [M+H]^+^ was *N*-benzyl-(9z,12z,15z)-octadecatrienamide. The collision-induced dissociation (CID) spectra of (Figure 3) were displayed as examples for the illustration of fragmentation patterns of macamides.

The five fragment ions (m/z 56, 96, 107, 136 and 260) which corresponded to butylene, (1Z, 4Z)-heptadecadiene, benzylamine, (1Z, 4Z, 7Z)-decatriene and (9Z, 12Z, 15Z)-octadecane-triene-ketone, respectively, through a classic α-cleavage in amide linkage, were detected in all standards and were considered as the diagnostic ions of macamides [2].

### 2.2. Mouse Leydig Cells Proliferation Activity of Ten Fractions

**Figure 4:**
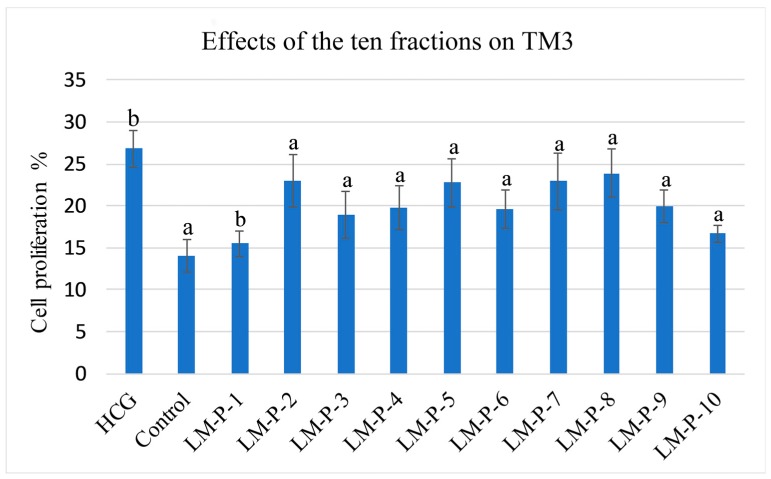
Effects of the ten fractions on TM3 (a *p* < 0.01, b *p* < 0.05).

The proliferation activity of these 10 fractions was assessed using the MTT assay in TM3. All the 10 fractions were found to possess the proliferation activity (Figure 4).

### 2.3. Screening of Active Compounds by Using Partial Least Squares

The selected initial data was further processed by PLS in order to establish a model for predicting the potential active components in *L. meyenii*. Parameters were set as follows: Confidence level was 95%, R2 = (0.0, 0.794), Q2 = (0.0, –0.285), and the parameters showed that the established PLS model was effective. We could use the PLS to carry on the weights analysis about the impact of the common peeks area exported from ten HPLC spectra of 10 fractions (x-axis) to the proliferative activity of TM3 (y-axis) and screening of major compounds which influenced bioactivity.

**Figure 5:**
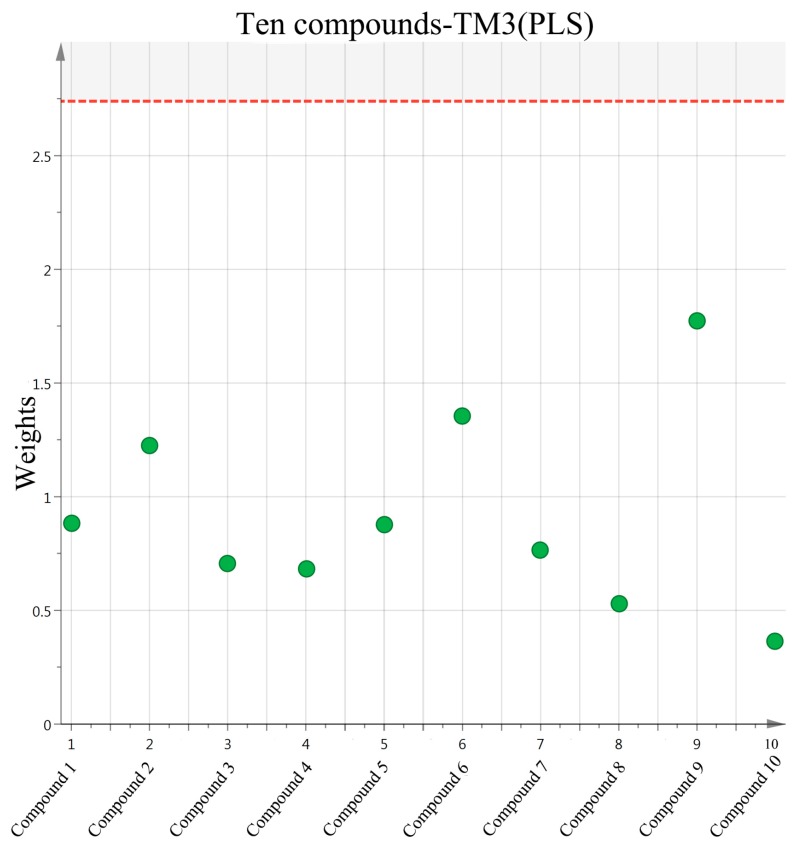
Model effect weights of the ten compounds on TM3.

In our data set, the weights plot summarized the variables both to explain X and to correlate to Y. The results were shown in (Figure 5). The weights greater than one indicated important variables, and three potential biological markers of *N*-benzyl-hexadecanamide (**9**), *N*-benzyl-(9z,12z)-octadecadienamide (**6**) and *N*-benzyl-(9z,12z,15z)-octadecatrienamide (**2**) had high contributions to the proliferation activity of TM3. Meanwhile, they were considered to be potential active compounds for further study [19].

### 2.4. Activity Evaluation of Active Components

The proliferation activity of the three compounds (**9**), (**6**), (**2**), were assessed using the MTT assay in TM3. All the compounds were found to possess proliferation activity.

**Figure 6:**
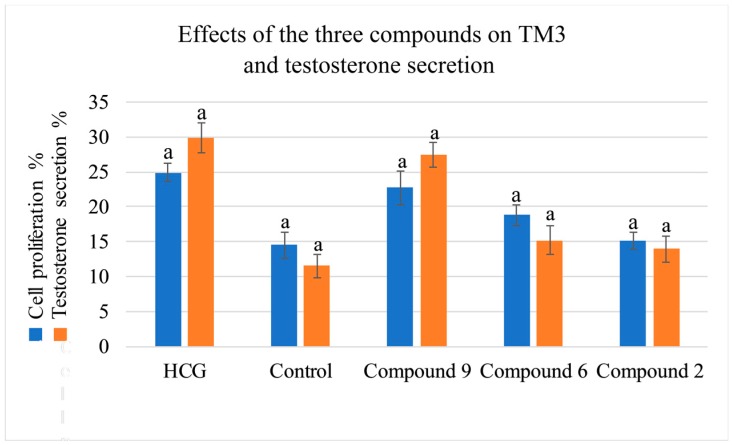
Effects of the three compounds on TM3 and testosterone secretion (a *p* < 0.01).

To better evaluate the improving sexual function of the three compounds (**9**), (**6**), (**2**), the testosterone secretion assay was tested, and the results were presented in a strong correlation between the values determined by the HPLC-DAD-MS2 method and that predicted by the testosterone secretion tested data was observed [20] (Figure 6).

### 2.5. Analysis of Molecular Docking

**Figure 7:**
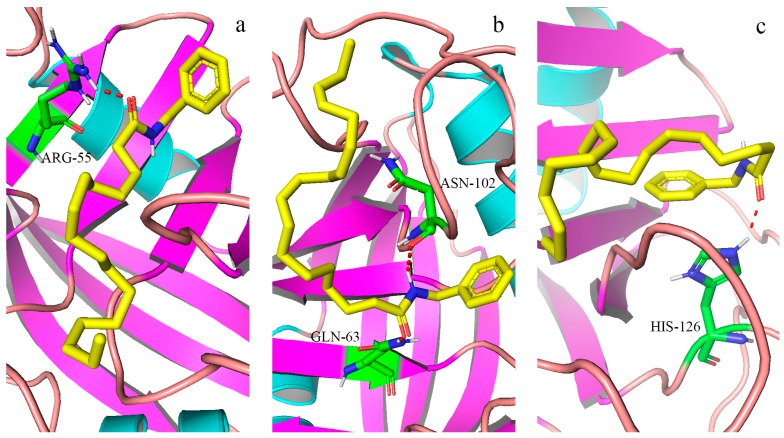
(**a**–**c**) Molecular docking of the three bioactive ligands with CypD, respectively. Ligands were shown in stick form and gray dashed lines were hydrogen bonds. The figure was prepared with PyMol. The interactions between bioactive ligands and binding sites were detailed in the article. (Figure 7a-(**9**), Figure 7b-(**6**), Figure 7c-(**2**)).

**Figure 8:**
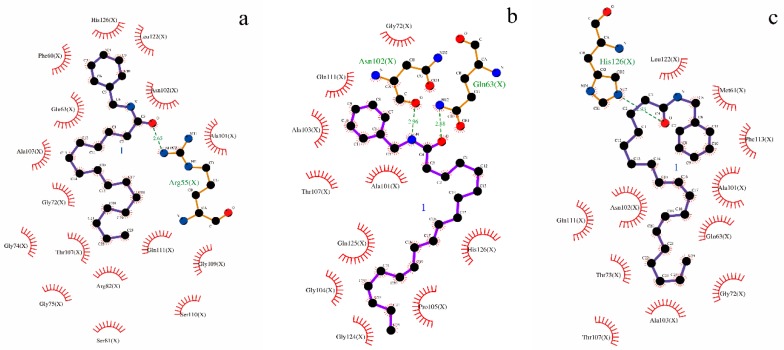
(**a**–**c**) Two-dimensional (2D) representation of hydrogen bond and hydrophobic interactions. Dashed lines represent hydrogen bonds, and spiked residues form hydrophobic interactions. (Figure 8a-(**9**), Figure 8b-(**6**), Figure 8c-(**2**))**.**

The molecular docking study further elucidated the binding mode of the three compounds at the active site of CypD. The binding pocket of CypD was large and shallow, consisting of residues Arg55, Ile57, Phe60, Met61, Gln63, Gly72, Thr73, Gly74, Ala101, Asn102, Phe113, Trp121, Leu122, and His126 etc [21]. In which it was known, four specific residues (Arg55, Gln63, Asn102 and Trp121) were involved in hydrogen bond interactions with CsA [12]. Molecular docking simulation revealed that ligands interacted with important amino acid residues surrounding the active site through plenty of interactions including hydrogen bond acceptor, hydrogen bond donor, hydrophobic interactions. The docked molecules interacted with essential amino acid forming proteins’ binding site. Unlike the case of full occupation by CSA, CypD- macamide complexes occupied only part of the binding pocket and might swing in the pocket [21]. (Figure 7a–c and Figure 8a–c). The lowest binding energy were found: −4.79 kcal/mol for (**9**), −4.55 kcal/mol for (**6**) and −4.18 kcal/mol for (**2**). The negative binding energy (G < 0) indicated that there were good binding affinity between the three compounds and CypD.

Normally, the interactions between CypD and the macamide were dependent on the structures of the macamides, as the number of hydrogen bonds and hydrophobic interactions increased, the affinity degree might increase, it was shown between (**6**) and (**2**). It was interesting that, a hydrogen bond was formed between residual Arg55 and N atoms of macamide, causing the electrons of the N atom to form a regular tetrahedron of Sp3 hybrid, with single-button rotation. A mutant CypD with a single amino acid substitution (Arg to Ala at position 55) that was predicted to produce a 1000-fold attenuation in isomerase activity failed to reverse the CsA effect [22]. Therefore, the lowest binding energy were found in (**9**).

The results showed there was a specific ligand-binding ability of macamide for CypD, which could be used in the inhibition of MPT pore opening, which caused mitochondrial damage. The homeostasis of the mitochondrial function ensured the maintenance of the StAR function, which was the first step in testosterone biosynthesis. CypD inhibitor could effectively bind CypD and inhibit the cis-trans isomerase activity of CypD, making the StAR expression stable, ultimately promoting testosterone secretion [23,24]. Therefore, one of the possible mechanisms of promoting testosterone secretion for thee compounds, which could be the bioactive markers of *L. meyenii*.

## 3. Materials and Methods

### 3.1. Materials

*Lepidium meyenii* was provided by Changchun University of Chinese Medicine and a voucher specimen (No. 201710) was deposited at the laboratory of Jilin Ginseng Academy, Changchun University of Chinese Medicine, P.R. China. Mouse leydig cells (TM3) were purchased from the Cell Bank of Type Culture Collection Chinese Academy of Sciences (Shanghai, China; cat. no. GNM24).

Standard compounds *N*-benzyl-hexadecanamide, *N*-benzyl-(9z,12z)-octadecadienamide and *N*-benzyl-(9z,12z,15z)-octadecatrienamide were provided by Yunnan Technical Center for Quality of Chinese Materia Medica (Yunnan, China). All standards were of purity greater than 98% and suitable for HPLC/MS/MS analysis.

### 3.2. Sample Preparation

The dried roots and rhizomes of *L. meyenii* (1000 g) were pulverized then sieved through a 20-mesh. The powder was extracted two times with 10 volumes of 95% ethanol (*v*/*v*) at 60 °C for 2 h. The filtrate was evaporated by a rotavapor at 60 °C and concentrated in vacuo to yield 24.32 g of brown residue. The residue (0.5 g) was further subjected to liquid-liquid partitioning to afford petroleum ether and water soluble extracts [25]. The resulting petroleum ether-soluble extract was applied to a silica gel column, and eluted with dichloromethane followed by (10:0 to 9:1, *v*/*v*) to give ten fractions (LM-P-1 to LM-P-10) [25]. The fraction was dissolved into 1 mL with acetonitrile and filtered with 0.22 μm filter membrane. The filtrate was used for HPLC analysis and testing of the proliferation of TM3.

### 3.3. High-Performance Liquid Chromatography-Photodiode Array Detector-Electrospray Ionization/Mass Spectrometry Method

An Agilent Technology 1100 Series HPLC system equipped with a quaternary pump, a degasser, a thermostatic auto-sampler and a photodiode array detector (DAD), was used for analysis (Agilent Technologies, Palo Alto, CA, USA). Chromatographic separations were carried out on a C18 analytical column Agilent Eclipse Plus-C18 (4.6 mm × 250 mm, 5μm) supplied by Agilent. The acetonitrile and water were used as the mobile phases (A) and (B), respectively, the optimized HPLC elution procedures were conducted as follows: 0–25 min, 80–90% (A); 25–70 min, 90–90% (A); 70–75 min, 90–100% (A). The flow-rate was 0.3 mL/min and the column temperature was maintained at 30 °C. The chromatogram was recorded at 210 nm. The injection volume of samples was 3.0 µL.

Agilent 1100 HPLC/MSD Trap mass spectrometer 6320 (Agilent) equipped with an electrospray ionization source was used in both positive and negative ion mode. An HPLC system coupled with DAD was controlled by an HPLC-MSD ChemStation software system. Auto MS2 mode of mass spectrometer was chosen to analyze the sample. The following operation parameters were used: capillary voltage: 4000 V; nebulizer pressure: 35 psi; drying gas: 9.0 L/min; gas temperature: 350 °C; skimmer voltage: 60 V. Liquid chromatography-electrospray ionization-mass spectrometry (LC-ESI-MS) accurate mass spectra were recorded across the range from 50 to 1200 m/z. The data recorded was processed with the Applied HPLC-MSD ChemStation software system [26] (1200, Agilent Technologies).

### 3.4. Cell Culture and Viability Assay

Mouse leydig cell (TM3) line is a mouse epithelial Leydig cell line. The TM3 cell line were grown in Dulbecco’s modified Eagle’s medium/F-12 nutrient mixture (DMEM/F-12) supplemented with 10% fetal bovine serum (Gibco; Thermo Fisher Scientific, Inc., Waltham, MA, USA), 1% penicillin (100 U/mL) and streptomycin (100 μg/mL) [27]. The human chorionic gonadotropin anhydrous (hCG) was obtained from Suolaibao Technology Co., Ltd. (Beijing, China).

Standard compounds *N*-benzyl hexadecanamide, *N*-benzyl-(9z,12z)-octadecadienamide and *N*-benzyl-(9z,12z,15z)-octadecatrienamide were dissolved in a culture medium containing a stock solution of 200 mg/L and further diluted to 62.5 μg/mL, 125 μg/mL, 250 μg/mL concentrations with culture medium containing 10% fetal bovine serum for 24 h. The DMEM/F12 concentrations (100 µL) was prepared as a control, and hCG concentrations (1 U/mL, 100 µL) were used as a positive control. Cells were cultured in a 37 °C incubator with 5% CO_2_ and 95% air [20]. The effects of fractions on Leydig cell viability were assessed by MTT (3-(4,5-dimethylthiazol-2-yl)-2,5-diphenyltetrazolium bromide) (Roche, Basle, Switzerland). The supernatant was collected to determine the testosterone levels using the Mouse Testosterone (T) ELISA kit (cat. no. JL10895; Shanghai Yuanye Biotechnology Co., Ltd., Shanghai, China), according to the manufacturer’s protocol.

### 3.5. Partial Least Squares Analysis and Statistical Analysis

The multivariate analysis of the acquired data was carried out by PLS using the SIMCA 11 software (Umetrics, Umea, Sweden). All assays were performed at least in triplicate and the results were expressed as a mean ± standard deviation (SD). The significant difference analysis was evaluated by one-way analysis of variance (ANOVA) test completed by the software of IBM SPSS Statistics 19 (International Business Machines Corp., New York, NY, USA). Significance was accepted at *p* < 0.05 [28].

### 3.6. Molecular Docking Studies

To further study the probable mechanism of the bioactive compounds with CypD, a molecular docking study which could conjecture the interactions of ligands within the constraint of receptors binding sites was performed in silico.

In the prediction, The X-ray crystal structure of CypD in the complex with its inhibitor CsA, 0.96 Å, was obtained from the Protein Data Bank (PDB ID: 2Z6W). The three-dimensional (3D) structures of the ligands were drawn and converted using ChemBioDraw Ultra and ChemBio 3D Ultra [29] (Cambridgesoft Corp., Waltham, MA, USA). The ligands and water molecules were removed from the crystal structure and the polar hydrogen was added by using AutoDock [30] (4.2.6, Department of Molecular Biology, The Scripps Research Institute, La Jolla, CA, USA).

Each grid computation was set up covering all the active sites where CsA was bounded. The grid was then concentrated on the center (80 Å, 40 Å, 80 Å, 0.375 Å, central coordinates x = –20.347, y = 13.119, and z = 11.232), respectively. The calculation of the docking score was repeated three times for each ligand. Fifty ligand−receptor complex conformations were generated for each test compound, in which the least building energy was considered for further analysis. Finally, PyMOL and LigPlot were used to present the docking results [31,32].

## 4. Conclusions

This work used the multivariate analysis to reveal some potential components, which improved sexual function from *L. meyenii*. We established an effective strategy based on HPLC-ESI-MS/MS with the PLS analysis for screening and determining the bioactive compounds which promote leydig cells proliferation and testosterone secretion. The 10 fractions were fractionated and their promoting activities on TM3 were demonstrated. With the aid of HPLC-ESI-MS/MS and the multivariate statistical software, the three potential improving sexual function markers were identified. Molecular docking was employed for further illustration in the mechanism of action for bioactivity.

In this study, correlation analysis was studied to explore the internal relationship between chemical constituents and pharmacological effects and discover the bioactive markers reflecting the traditional efficacy of *L. meyenii*. The results specified the three compounds as potential bioactive markers could lay a foundation for the improvement of quality standard of *L. meyenii*.

## Figures and Tables

**Table 1:** HPLC- ESI-MS data of 10 common peeks.

No.	Retention Time (min)	UV Absorption Characteristics λmax (nm)	Observed m/z	Fragment Ion	Compound Structure	Component Name
**1**	35.3	210	398	138,261	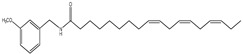	*N*-(3-methoxybenzyl)-(9zN,12zN,15z)- octadecatrienamide
**2**	36.4	210	368	108,232, 261,272	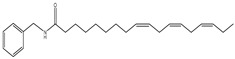	*N*-benzyl-(9z,12z,15z)-octadecatrienamide
**3**	37.7	210	368	108,261, 285	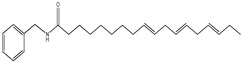	*N*-benzyl-(9E,12E,15E)- octadecatrienamide
**4**	38.7	210	368	108,261	Unknown	Unknown
**5**	44.8	210	400	138,263, 302	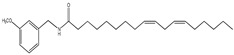	*N*-(3-methoxybenzyl)-(9Z,12Z)-octadecadienamide
**6**	46.6	210	370	108,232, 263,272	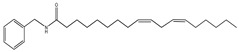	*N*-benzyl-(9z,12z)-octadecadienamide
**7**	48.9	210	370	108,263	Unknown	Unknown
**8**	50.3	210	370	108,263, 285	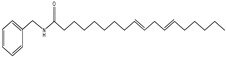	*N*-benzyl-(9E,12E)-octadecadienamide
**9**	59.6	210	346	108,239, 268,268	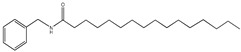	*N*- benzyl- hexadecanamide
**10**	63.0	210	372	108,165	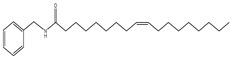	*N*-benzyl-9Z-octadecenamide
